# ISLAND: in-silico proteins binding affinity prediction using sequence information

**DOI:** 10.1186/s13040-020-00231-w

**Published:** 2020-11-25

**Authors:** Wajid Arshad Abbasi, Adiba Yaseen, Fahad Ul Hassan, Saiqa Andleeb, Fayyaz Ul Amir Afsar Minhas

**Affiliations:** 1 Computational Biology and Data Analysis Laboratory, Department of Computer Science and Information Technology, King Abdullah Campus, University of Azad Jammu & Kashmir, Muzaffarabad, Pakistan; 2 Biomedical Informatics Research Laboratory, Department of Computer and Information Sciences, Pakistan Institute of Engineering and Applied Sciences (PIEAS), Nilore, Islamabad Pakistan; 3 Biotechnology Laboratory, Department of Zoology, King Abdullah Campus, University of Azad Jammu & Kashmir, Muzaffarabad, Pakistan; 4 Department of Computer Science and the PathLAKE Consortium, University of Warwick, Coventry, UK

**Keywords:** Protein sequence analysis, Protein-protein interaction, Support vector machines, Web services, Binding affinity

## Abstract

**Background:**

Determining binding affinity in protein-protein interactions is important in the discovery and design of novel therapeutics and mutagenesis studies. Determination of binding affinity of proteins in the formation of protein complexes requires sophisticated, expensive and time-consuming experimentation which can be replaced with computational methods. Most computational prediction techniques require protein structures that limit their applicability to protein complexes with known structures. In this work, we explore sequence-based protein binding affinity prediction using machine learning.

**Method:**

We have used protein sequence information instead of protein structures along with machine learning techniques to accurately predict the protein binding affinity.

**Results:**

We present our findings that the true generalization performance of even the state-of-the-art sequence-only predictor is far from satisfactory and that the development of machine learning methods for binding affinity prediction with improved generalization performance is still an open problem. We have also proposed a sequence-based novel protein binding affinity predictor called ISLAND which gives better accuracy than existing methods over the same validation set as well as on external independent test dataset. A cloud-based webserver implementation of ISLAND and its python code are available at https://sites.google.com/view/wajidarshad/software.

**Conclusion:**

This paper highlights the fact that the true generalization performance of even the state-of-the-art sequence-only predictor of binding affinity is far from satisfactory and that the development of effective and practical methods in this domain is still an open problem.

## Background

Protein binding affinity is a key factor in enabling protein interactions and defining structure-function relationships that drive biological processes [1]. Accurate measurement of binding affinity is crucial in understanding complex biochemical pathways and to uncover protein interaction networks. It is also measured as part of drug discovery and design to improve drug specificity [2]. It can be measured in terms of the disassociation constant (*K*_*d*_) through different experimental methods such as Nuclear magnetic resonance spectroscopy, gel-shift and pull-down assays, analytical ultracentrifugation, Surface Plasmon Resonance (SPR), spectroscopic assays, etc [3, 4]. However, the accuracy of these methods depends on dissociation rates and these methods cannot be applied at a large scale due to cost and time constraints [3, 5]. Therefore, accurate computational techniques can play an important role in the affinity determination of protein complexes.

Various computational methods for binding affinity prediction have been proposed based on free energy perturbation, empirical scoring, and force-field potentials [6–12]. These scoring function based methods are typically trained and evaluated on limited datasets. These methods fail to accurately predict binding affinities for diverse datasets [13].

**Fig. 1:**
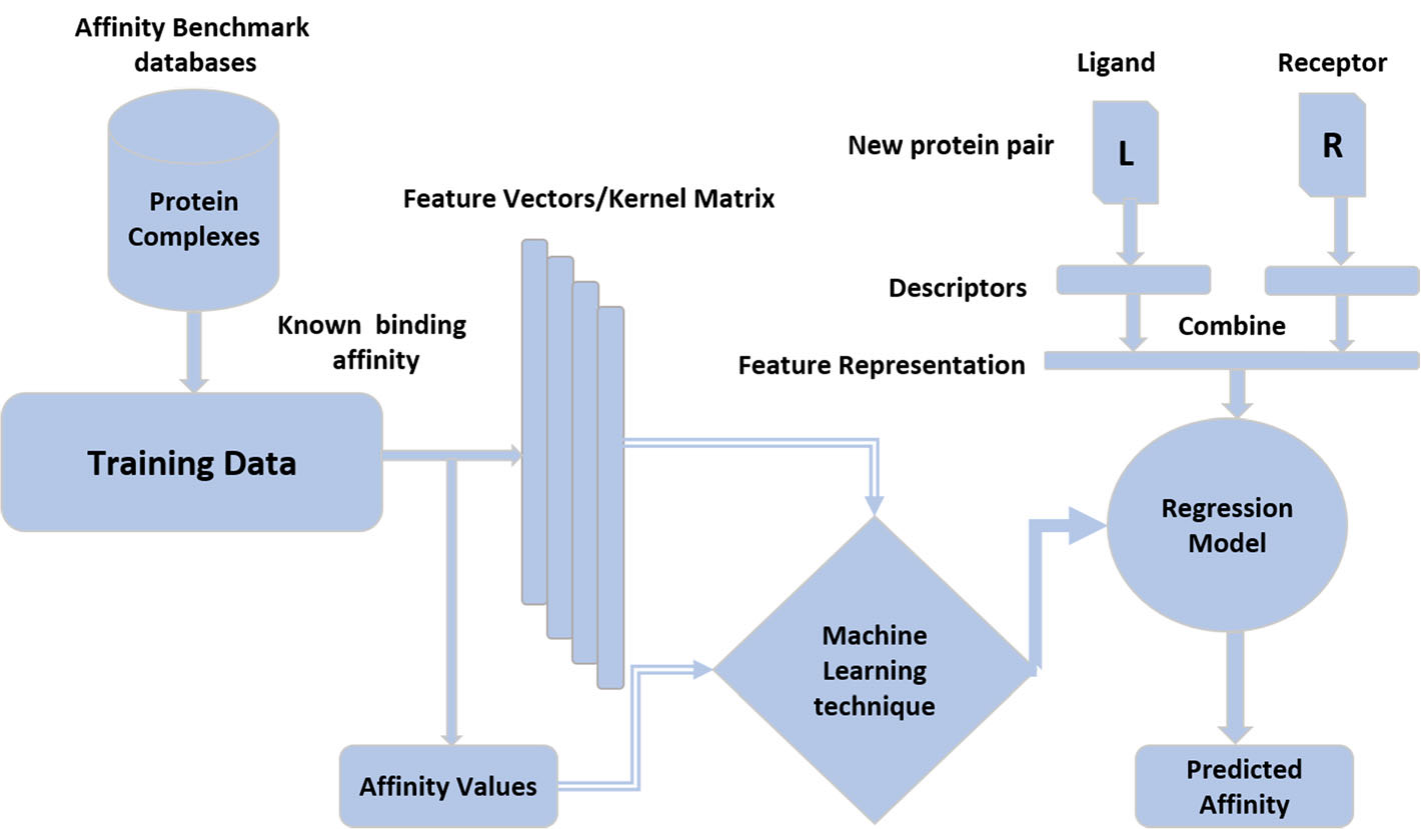
A general framework for protein affinity prediction using machine learning techniques

Among computational binding affinity prediction methods, machine learning is preferred because of its implicit treatment of any relevant factors involved in protein-protein interactions (PPIs) and the flexibility of using empirical data instead of a fixed or predetermined function form [14]. A representation of the design and use of machine learning models for binding affinity prediction is given in Fig. 1. Machine learning based affinity prediction models require a dataset of diverse protein complexes with experimentally determined affinity values for training. By extracting the feature representation of protein complexes, a regression model is trained which can be used for affinity prediction of a novel complex (Fig. 1). A number of machine learning based studies for protein binding affinity prediction have been proposed in the literature [5, 15–19]. Most of these studies are based on a protein binding affinity benchmark dataset with 3-D structures of 144 protein complexes [20]. The affinity prediction models proposed by Moal et al.*,* Tian et al.*,* and Vangone and Bonvin in their studies are based on 3-D protein structures [5, 15, 16]. However, protein structures are not available for most protein complexes. Consequently, the sequence-based prediction of binding affinity is an important research problem. Sequence-based binding affinity prediction is challenging because proteins interaction and binding affinity are dependent upon protein structures and functions.

Among sequence-based protein binding affinity prediction models using protein binding affinity benchmark dataset, the model proposed by Yugandhar and Gromiha (PPA-Pred2) is the state of the art absolute binding affinity predictor [17]. PPA-Pred2 claims high accuracy with a high correlation score between true and predicted binding affinity values [21]. However, their proposed model performed poorly on an external validation dataset [22]. Furthermore, their prediction errors are, surprisingly, lower than the reported deviation in experimental measurements of binding affinity values and the error rates of structure-based prediction techniques [20, 22]. Yugandhar and Gromiha have attributed this issue to the difference in experimental conditions and computational platforms [21]. In this work, we have replicated the validation of PPA-Prep2 on an external independent test dataset as performed by Moal *et. al.* [22]. Moreover, protein binding affinity prediction models proposed by Chen M, et al. and Srinivasulu YS, et al., had not been evaluated using any external validation datasets, and also these studies did not provide an interface to perform such a validation [18, 19]. These simple researches have highlighted the need to revisit sequence-based binding affinity prediction and develop novel predictors that can be used in a practical setting. To address this, we have proposed a new binding affinity prediction model called ISLAND (In SiLico protein AffiNity preDictor). Our proposed model uses sequence features alone and gives higher prediction accuracy than the PPA-Pred2 web server.

## Methods

In this section, we have discussed in detail the methodology adopted to develop and evaluate the performance of sequence-based protein binding affinity predictors.

### Datasets and preprocessing

We have used protein binding affinity benchmark dataset 2.0 for evaluation of PPA-Pred2 webserver and development of the proposed method ISLAND [20]. This dataset contains 144 non-redundant complexes of proteins for which both bound and unbound structures of the ligand and receptor proteins are available. Protein binding affinities are given in terms of binding free energy (**∆*G***) and disassociation constant (***K***_***d***_). The binding free energy (**∆*G***) ranges from − 18.58 to − 4.29. Following the same data curation and preprocessing technique used by Yugandhar and Gromiha, we have selected 135 complexes from this dataset [17]. This allows us to have a direct comparison of our method with the one proposed by Yugandhar and Gromiha [17].

We have also used an external independent test dataset of 39 protein-protein complexes with known binding free energy (**∆G**) to perform a stringent test of performance comparison between PPA-Pred2 and ISLAND. This dataset is derived from Chen et al. by removing complexes having more than two chains, involving chains of size less than 50 residues, and having an overlap with training data [23]. This dataset has also been used by Moal *et. al.* in their evaluation of binding affinity prediction techniques [22].

### Evaluation of the PPA-Pred2 webserver

In order to investigate the accuracy of PPA-Pred2, we evaluated its performance on the selected dataset. For this purpose, we accessed PPA-Pred2 [17] through its webserver (URL: http://www.iitm.ac.in/bioinfo/PPA_Pred/) on 03-02-2017. This webserver takes amino acid sequences of ligand and receptor of a protein complex and returns predicted values of change in binding free energy (**∆*G***) and disassociation constant (***K***_***d***_) [17]. The results obtained through this evaluation will also serve as a baseline in this study.

### Sequence homology as affinity predictor

In order to confirm whether simple homology is enough to predict protein binding affinity accurately or not, we have developed a sequence homology-based protein binding affinity predictor as a baseline. For this purpose, we predicted the affinity value of a query protein complex based on the affinity value of its closest homolog in our dataset of protein complexes with known binding affinity values. We performed the Smith-Waterman alignment to determine the degree of homology between two protein complexes using BLOSUM-62 substitution matrix with gap opening and extension penalties of − 11 and − 1, respectively [24, 25].

### Proposed methodology

We have developed a sequence-only regression model called ISLAND (In SiLico protein AffiNity preDictor), to predict absolute protein binding affinity values rather calssifying protein complexes into low and high affinity as in case of LUPI [26]. To develop ISLAND, we have used different regression methods, evaluation protocols, and sequence-based feature extraction techniques. The methodology adopted for the development of the ISLAND is detailed below.

### Sequence-based features

In machine learning based prediction models, we require a feature representation of each example for training and testing (Fig. 1). Therefore, we have represented each complex in our dataset through a feature representation obtained from individual chains in the ligand (***l***) and receptor (***r***) of each complex. We used several explicit features and various kernel representations to model sequence-based attributes of protein complexes. We discuss the sequence-based features used in this study below.

#### Explicit features

##### Amino acid composition features (AAC)

These features capture the occurrences of different amino acids in a protein sequence. It gives a 20-dimensional feature vector ***ϕ***_***AAC***_(***s***) of a given sequence ***s*** such that the ***ϕ***_***AAC***_(***s***)_***k***_ contains the number of times amino acid ***k*** occurs in ***s*** [27]. This feature representation has successfully been used to predict protein interactions, binding sites, and prion activity [27–29].

##### Average BLOSUM-62 features (Blosum)

In contrast to AAC, this feature representation models the substitutions of physiochemically similar amino acids in a protein. In this feature representation, protein sequence ***s*** is converted into a 20-dimensional feature vector by simply averaging the columns from the BLOSUM-62 substitution matrix corresponding to the amino acids in the given sequence. Mathematically, ${\boldsymbol{\phi}}_{\boldsymbol{B}\boldsymbol{losum}}\left(\boldsymbol{s}\right)=\frac{\mathbf{1}}{\left|\boldsymbol{s}\right|}{\sum}_{\boldsymbol{i}=\mathbf{1}}^{\left|\boldsymbol{s}\right|}{\boldsymbol{B}}_{\boldsymbol{i}}$, where ***B***_***i***_ is the column of the BLOSUM-62 substitution matrix [24] corresponding to the i^th^ residue in ***s***.

##### Propy features (propy)

In order to capture the biophysical properties of amino acids and sequence-derived structural features of a given protein sequence, we used a feature extraction package called propy [30]. It gives a 1537-dimensional feature representation ***ϕ***_***propy***_(***s***) of a given sequence ***s***. This representation includes pseudo-amino acid compositions (PseAAC), autocorrelation descriptors, sequence-order-coupling number, quasi-sequence-order descriptors, amino acid composition, transition and the distribution of various structural and physicochemical properties [31, 32].

##### Position specific scoring matrix features (PSSM)

This feature representation models the evolutionary relationships between proteins. To get this representation, we used the Position Specific Scoring Matrix (PSSM) of a given protein sequence [33]. We obtained the PSSM for each protein chain in a complex by using PSI-BLAST for three iterations against the non-redundant (nr) protein database with an e-value threshold of **10**^**−3**^ [33, 34]. In this feature representation, we represent the protein sequence ***s*** by the average of columns in its PSSM. This results in a 20-dimensional feature vector ${\boldsymbol{\phi}}_{\boldsymbol{PSSM}}\left(\boldsymbol{s}\right)=\frac{\mathbf{1}}{\left|\boldsymbol{s}\right|}{\sum}_{\boldsymbol{i}=\mathbf{1}}^{\left|\boldsymbol{s}\right|}{\boldsymbol{F}}_{\boldsymbol{i}}^{\boldsymbol{s}}$, where ${\boldsymbol{F}}_{\boldsymbol{i}}^{\boldsymbol{s}}$ is the column in the PSSM corresponding to the i^th^ residue in *s*.

##### ProtParam features (ProtParam)

In order to capture different physiochemical properties of a protein such as the molecular weight of the protein, aromaticity, instability index, isoelectric point, and secondary structure fractions, we have used ProParam ExPASy tools to get ProtParam representation [35–37]. This leads to a 7-dimensional feature representation ***ϕ***_***ProtParam***_(***s***) of a given sequence ***s***.

#### Kernel representations

In addition to using explicit protein sequence features in our machine learning models for binding affinity prediction, we have also experimented with different sequence-based kernel [38, 39]. Kernel methods present an alternate way of sequence representation by modeling the degree of similarity between protein sequences instead of an explicit feature representation [38]. Kernel-based methods such as support vector machines and support vector regression can make use of these kernel function scores in their training and testing [40]. Different sequence kernels used in this work are described below. Each of these kernels ***k***(***a***, ***b***) can be interpreted as a function that measures the degree of similarity between sequences ***a*** and ***b***.

##### Smith-Waterman alignment kernel (SW kernel)

We have used the Smith-Waterman alignment algorithm for determining the degree of similarity between two protein sequences [25]. The Smith-Waterman kernel ***k***_***SW***_(***a***, ***b***) is simply the alignment score obtained from the Smith-Waterman local alignment algorithm using BLOSUM-62 substitution matrix with gap opening and extension penalties of − 11 and − 1, respectively. It is important to note that this kernel may not satisfy the Mercer’s conditions as the eigen values of the kernel matrix may be negative [41]. We addressed this issue by subtracting the most negative eigenvalue of the original kernel matrix from its diagonal elements [42]. From a theoretical point of view, this kernel can be interpreted as the optimal local alignment score of the two sequences [42]. Mathematically, the Smith-Waterman alignment score ***k***_***SW***_(***a***, ***b***) between sequences, ***a*** and ***b*** can be written as follows [42].

1 $$ {\boldsymbol{k}}_{\boldsymbol{SW}}\left(\boldsymbol{a},\boldsymbol{b}\right)={\boldsymbol{\max}}_{\boldsymbol{\pi} \in \boldsymbol{\Pi} \left(\mathbf{l},\mathbf{r}\right)}\boldsymbol{p}\left(\boldsymbol{a},\boldsymbol{b},\boldsymbol{\pi} \right) $$

Here, **Π**(**a**, **b**) denote the set of all possible local alignments between ***a*** and ***b***, and ***p***(***a***, ***b***, ***π***) represents the score of the local alignment ***πϵΠ***(***a***, ***b***) between ***a*** and ***b***.

##### Local alignment kernel (LA kernel)

Local alignment kernel is useful for comparing sequences of different lengths that share common parts [40, 42]. In contrast to the Smith-Waterman alignment kernel which considers only the optimal alignment, this kernel sums up contributions of all the possible local alignments of input sequences. Mathematically, the local alignment score *k*_*LA*_(*a*, *b*) between sequences, *a* and *b* can be written as follows [42].

2 $$ {k}_{LA}^{\beta}\left(a,b\right)={\sum}_{\pi \in \Pi \left(\mathrm{a},\mathrm{b}\right)}\exp \left(\beta p\left(a,b,\pi \right)\right) $$

Here in Eq. (2), *β* ≥ 0 is a parameter that controls the sensitivity of the LA kernel. For larger values of *β* score of LA kernel approaches SW kernel score [42]. We have used *β* = 0.1 based on empirical performance.

##### Mismatch kernel (MM kernel)

The mismatch kernel captures the degree of overlap between subsequences of the two sequences while allowing mismatches [43]. MM kernel ${k}_{MM}^{k,m}\left(a,b\right)$ gives the number of subsequences of length *k* that are present in both the input sequences *a* and *b* with a maximum of *m* mismatches. Ranges for the values of *k* and *m* are 3 − 9 and 0 − 5, respectively. We have used *k* = 5 and *m* = 3 based on empirical performance.

### Complex level features representation

**Fig. 2:**
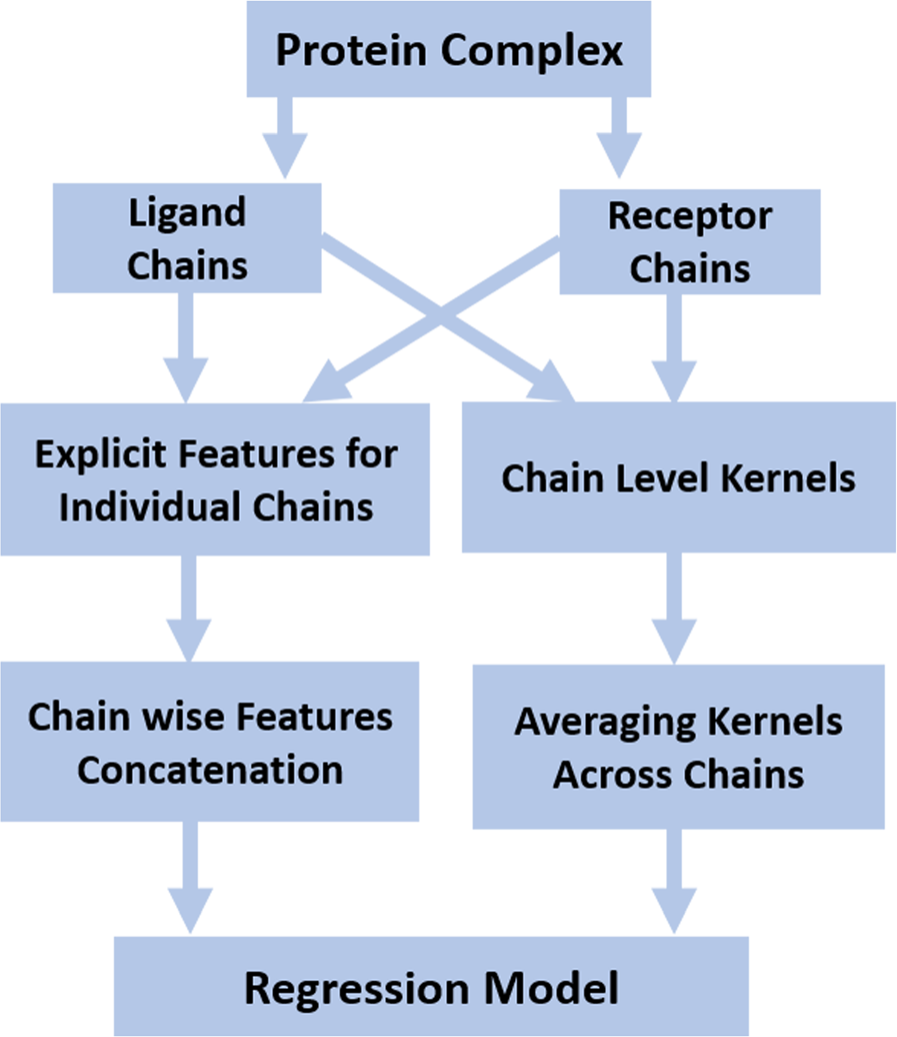
Techniques adopted for generating sequence-based feature representation of a protein complex for developing machine learning based protein binding affinity prediction models

We need to predict protein binding affinity at the complex level. Since we have extracted features at the chain level, therefore, we require a mechanism to obtain a complex level feature representation from individual chains. The basic mechanism of combining individual chain level feature representation from each ligand and receptor to form a complex level representation is shown in Fig. 2. Complex level representation is obtained for explicit features by concatenation of chain level features and for kernels by adding kernels over the constituent chains of a complex.

#### Feature concatenation

In our machine learning model, a complex *c* is represented by the tuple *c* ≡ ((*l*, *r*), *y*), where (*l*, *r*) is the pair of ligand and receptor proteins in the complex and *y* is the corresponding affinity value. To generate the complex level feature representation ***ψ***(*c*), we simple concatenate the feature representations of respective ligand and receptor as $$ \boldsymbol{\psi} (c)=\left[\begin{array}{c}{\boldsymbol{\psi}}_{Avg}(l)\ \\ {}{\boldsymbol{\psi}}_{Avg}(r)\end{array}\right] $$. Here, ${\boldsymbol{\psi}}_{Avg}(l)=\frac{1}{\left|l\right|}{\sum}_{q\in l}\boldsymbol{\phi} (q)$ and ${\boldsymbol{\psi}}_{Avg}(r)=\frac{1}{\left|r\right|}{\sum}_{q\in r}\boldsymbol{\phi} (q)$ are the explicit feature representations averaged across all the chains present in the ligand and receptor proteins, respectively. This method of feature representation generation has already been used for protein interacting residues predictor [44].

#### Combining kernels

To make predictions at the complex level from sequence-based kernels, we have developed a complex-level kernel by simply averaging the kernel function values of individual chains from the two complexes [38]. Mathematically, the kernel over complexes *c* and *c*^′^ is given by $K\left(c,{c}^{\prime}\right)=\frac{1}{\left|c\right|\times \left|{c}^{\prime}\right|}\sum \limits_{q\in c,{q}^{\prime}\in {c}^{\prime\prime }}k\left(q,{q}^{\prime}\right)$, where *k*(*q*, *q*^′^) is the chain level kernel over two chains from the two complexes.

### Regression models

Here, we begin by presenting the binding affinity prediction problem as a regression problem. In machine learning based affinity prediction, a dataset consisting of *N* examples (*c*_*i*_, *y*_*i*_), where *i* = 1…*N*. In this representation, *c*_*i*_ is a complex with known binding affinity *y*_*i*_. The feature representation of *c*_*i*_ is ***ψ***(*c*_*i*_). Our objective in machine learning based regression is to train a model *f*(*c*) that can predict the binding affinity of the complex *c*. The learned regression function *f*(∙) should generalize well over previously unseen complexes. We used the following regression techniques through Scikit-learn to get different regression models [45]. It is also important to note that the feature representations are normalized to have unit norm and standardized to zero mean and unit standard deviation before using them in the regression model.

#### Ordinary least-squares regression (OLSR)

Ordinary least squares (OLS) estimates the regression function *f*(*c*) = ***w***^*T*^***ψ***(*c*) + *b* by minimizing the sum of squared error between the actual and predicted affinity values ${\min}_{\boldsymbol{w},b}\sum \limits_i^N{\left({y}_i-f\left({\boldsymbol{c}}_i\right)\right)}^2$ [46]. Here, ***w*** and *b* are parameters to be learned. This technique has been used previously for protein binding affinity prediction [17]. We have used this technique as a baseline in our study.

#### Support vector regression (SVR)

Support Vector Machines have been effectively used to solve different computational problems in bioinformatics [47]. Support Vector Regression (SVR) performs regression using *ε*-insensitive loss and, by controlling model complexity [48]. Training a SVR for protein binding affinity prediction involves optimizing the objective function given in Eq. (3) to learn a regression function *f*(*c*) = ***w***^*T*^***ψ***(*c*) + *b*.

$$ {\mathit{\min}}_{w,b}\frac{1}{2}{\left\Vert \boldsymbol{w}\right\Vert}^2+\mathrm{C}\sum \limits_{i=1}^N\left({\xi}_i^{+}+{\xi}_i^{-}\right) $$

3 $$ Such\ that\ for\  all\ i:\left\{\begin{array}{c}\begin{array}{c}{y}_i-f\left({\boldsymbol{c}}_i\right)\le \varepsilon +{\xi}_i^{+}\\ {}f\left({\boldsymbol{c}}_i\right)-{y}_i\le \varepsilon +{\xi}_i^{-}\end{array}\\ {}{\xi}_i^{+},{\xi}_i^{-}\ge 0\end{array}\right. $$

Here, $\frac{1}{2}{\left\Vert \boldsymbol{w}\right\Vert}^2$ controls the margin, ${\xi}_i^{+}$ and ${\xi}_i^{-}$ capture the extent of margin violation for a given training example and *C* is the penalty of such violations [47]. We used both linear and radial basis function (rbf) SVR in this study. The values of C, gamma, and epsilon were optimized during model selection. SVR has already been used for the same purpose in previous studies [17].

#### Random Forest regression (RFR)

Random Forest regression (RFR) is an ensemble of regression trees used for nonlinear regression [49]. Each regression tree in the RF is based on randomly sampled subsets of input features. We optimized RF with respect to the number of decision trees and a minimum number of samples required to split in this study using grid search. This regression technique has been used in many related studies [15, 50, 51].

### Model evaluation and performance assessment

To evaluate the performance of all the trained regression models, we have used Leave One Complex Out (LOCO) cross-validation (CV) [52]. In LOCO, a regression model is developed with (*N* – 1) complexes and tested on the left out complex. This process is repeated for all the *N* complexes present in the dataset. We used Root Mean Squared Error $\mathrm{RMSE}=\sqrt{\frac{1}{n}{\sum}_{i=1}^N{\left({y}_i-f\left({c}_i\right)\right)}^2}$ and Pearson correlation coefficient (*P*_*r*_) between the predicted *f*(*c*_*i*_) and actual *y*_*i*_, as performance measures for model evaluation and performance assessment. To check the statistical significance of the results, we have also estimated the *P*-value of the correlation coefficient scores. We used grid search over training data to find the optimal values of hyper-parameters of different regression models.

### Webserver

We have deployed ISLAND as a webserver that takes a pair of protein sequences in plain text and predicts their binding affinity. After the successful submission of protein sequences, the result page shows predicted binding affinity along with disassociation constant (*K*_*d*_). A Python implementation of the proposed method together with a webserver is available at http://faculty.pieas.edu.pk/fayyaz/software.html# island.

## Results and discussion

In this section, we discuss the results and give details of the major outcomes of our study.

### Binding affinity prediction through sequence homology

As a baseline, we have obtained the predicted affinity values of all 135 complexes in our dataset using a sequence homology-based affinity prediction method. The Pearson correlation coefficient (*P*_*r*_) between predicted and experimental values of *∆G* is 0.29 with a Root Mean Squared Error (*RMSE*) of 3.20. These results with poor correlation and high RMSE value show that the sequence homology only cannot be effectively used to predict the binding affinity of the protein complexes. As discussed in the next section, our machine learning based method performs significantly better than homology-based predictions.

### Binding affinity prediction through ISLAND

We have evaluated the performance of three different regression models (OLSR, RFR, and SVR) along with eight different types of sequence descriptors with LOCO cross-validation over the docking benchmark dataset. The results of this analysis are shown in Table 1 in the form of Root Mean Squared Error (*RMSE*) and Pearson correlation coefficient (*P*_*r*_) along with statistical significance (*P*-value). With explicit features, we obtained a maximum correlation of 0.41 with RMSE = 2.60 between predicted and experimental values of *∆G* using propy through SVR (Table 1). While using kernel descriptors, we obtained a maximum correlation of 0.44 with an RMSE = 2.56 between predicted and experimental *∆G* values using the local alignment kernel (see in Table 1). We have achieved the best performance through local kernel across all sequence descriptors used in this study as shown in Table 1. Moreover, LA kernel performs better than SW kernel because of considering the effect of all the local alignments rather taking the best alignment as in the case of SW kernel. The RMSE value of ISLAND predictions is quite close to the range of experimental uncertainties (1–2 kcal/mol) as reported by Kastritis et al. [20]. Our proposed method outperforms the previous sequence-based method proposed by Srinivasulu YS, et al., with a reported correlation coefficient of 0.34 through Jackknife cross validation [19]. Another protein sequence-based method involving deep learning proposed by Chen M, et al., reported a higher accuracy with a correlation coefficient of 0.873 using 10-fold cross-validation and SKEMPI dataset [18, 53]. However, the cross-validation scheme adopted by Chen M, et al., may not conform to the underlying problem as SKEMPI dataset involves more than one mutant proteins of a single protein complex [18, 52, 53].

**Table 1:** Performance of regression models trained on the range of protein sequence descriptors using loco cross validation

Feature Descriptors	Regression Models								
	OLSR			RFR			SVR		
	P_**r**_	***P***-value	RMSE	P_**r**_	***P***-value	RMSE	P_**r**_	***P***-value	RMSE
AAC	0.20	1.5 × 10^− 2^	3.19	0.40	6.4 × 10^− 7^	2.66	0.40	1.0 × 10^−6^	2.69
Blosum	0.20	1.4 × 10^−2^	3.10	0.37	2.8 × 10^−7^	2.71	0.39	1.5 × 10^−5^	2.67
propy	0.14	1.3 × 10^−1^	3.67	0.39	3.0 × 10^−3^	2.64	0.41	1.1 × 10^−6^	2.60
PSSM	0.19	7.2 × 10^−1^	3.68	0.38	1.1 × 10^−5^	2.67	0.37	1.5 × 10^−5^	2.66
ProtParam	0.25	3.0 × 10^−3^	2.82	0.34	4.7 × 10^−5^	2.72	0.37	9.4 × 10^−6^	2.64
SW kernel	results not applicable						0.37	2.1 × 10^−6^	2.63
LA kernel							**0.44**	**1.2 × 10**^**−8**^	**2.56**
MM kernel							0.38	7.1 × 10^−6^	2.66

The performance of the ISLAND is also comparable with the methods based on 3-D protein structures such as DFIRE (*P*_*r*_ = 0.35), PMF (*P*_*r*_ = 0.37), RBF (*P*_*r*_ = 0.44), M5’ (*P*_*r*_ = 0.45) and RF (*P*_*r*_ = 0.48) as reported by Moal et al. [15]. Despite getting the comparable performance of ISLAND with structure-based methods, there is still a lot of room for improvement in affinity prediction from sequence information alone.

### Comparison using external independent test dataset

**Fig. 3:**
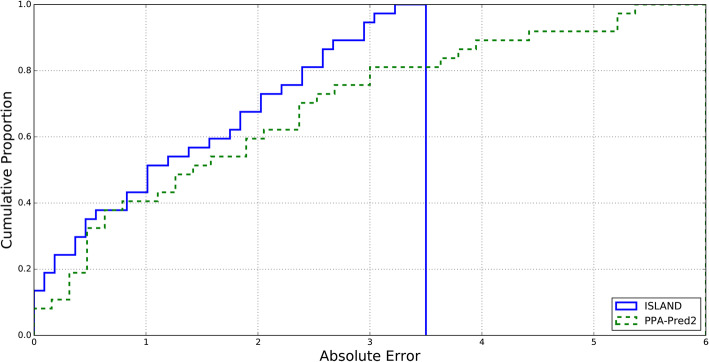
Cumulative histogram of absolute error between actual and predicted binding affinity values through ISLAND and PPA-Pred2 on external independent validation dataset

We obtained the predicted binding affinity values for all the complexes in our external validation dataset using both PPA-Pred2 and ISLAND. We have seen a significant performance improvement of the ISLAND in terms of RMSE between predicted and experimental *∆G* values. We obtained an RMSE of 1.98 with ISLAND whereas PPA_Pred2 gives us an RMSE of 4.78. We have also seen a significant performance improvement of both the methods in terms of Pearson correlation coefficient and absolute error with values 0.35, 1.52 and 0.05, 2.63 through ISLAND and PPA_Pred2, respectively. We have also shown a comparison between ISLAND and PPA-Pred2 in terms of absolute error between predicted and actual binding affinity values of all the complexes in our validation set in Fig. 3. The binding affinity of > 60% complexes were predicted within an absolute error of 1.5 kcal/mol using ISLAND, whereas, through PPA-Pred2 absolute error for these complexes is above 2.5 kcal/mol (see in Fig. 3). These results show better performance of our proposed method for binding affinity prediction of proteins in a complex in comparison to PPA-Pred2. These performance improvements of ISLAND over PPA-Pred2 are based on a proper model selection with parameters tuned using grid search and better feature engineering by using different kernels. Moreover, these results also support the criticism of Moal *et. at*., on PPA-Pred2 and suggest a need for further work on methods of protein binding affinity prediction using sequence information [22].

## Conclusions

This paper highlights the fact that the true generalization performance of even the state-of-the-art sequence-only predictor of binding affinity is far from satisfactory and that the development of effective and practical methods in this domain is still an open problem. As already suggested in recent studies by Dias & Kolaczkowski and Abbasi et al., to achieve better performance in this domain, we need either a significant increase in the amount of quality affinity data or methods of leveraging data from similar problems [26] [54]. We also propose a novel sequence-only predictor of binding affinity called ISLAND which gives better accuracy than PPA-Pred2 webserver and other existing methods over the same external independent test set.

## Data Availability

All data generated or analyzed during this study are included in this paper or available at online repositories. A Python implementation of the proposed method together with a webserver is available at https://sites.google.com/view/wajidarshad/software and https://github.com/wajidarshad/ISLAND.
